# Is changing the postoperative pain management in total knee arthroplasty from femoral nerve block to local infiltration analgesia successful? Retrospective trial with the first and last 100 patients

**DOI:** 10.1186/s13018-020-01981-3

**Published:** 2020-10-19

**Authors:** Michael Najfeld, Robert Hube, Ann-Kathrin Kujat, Hermann Otto Mayr, Kathi Thiele

**Affiliations:** 1 OCM Orthopädische Chirurgie München, Steinerstr. 6, 81369 Munich, Germany; 2 Centrum für Muskuloskeletale Chirurgie, Campus Mitte Universitätsmedizin Berlin, Berlin, Germany; 3 Klinik für Orthopädie und Traumatologie, Universität Freiburg, Freiburg, Germany; 4 Klinik für Knie, Hüfte und Schulterchirurgie, Schön Klinik München Harlaching, Munich, Germany

**Keywords:** Local infiltration analgesia, Arthroplasty, Replacement, Knee, Nerve block, Pain

## Abstract

**Purpose:**

In recent years, there has been an increasing interest in local infiltration analgesia (LIA) as a technique to control postoperative pain. We compared this technique to the gold standard the 3 in 1 femoral nerve block (FNB) in postoperative pain management after total knee arthroplasty (TKA) in a large patient population. This trial analyzes in the early postoperative phase the pain, range of motion, and consumption of pain medications after TKA.

**Methods:**

We conducted a retrospective trial that included all patients who were undergoing primary TKA by one single surgeon in a high-volume arthroplasty center in 2015. Patients who have secondary osteoarthritis due to rheumatoid arthritis or previous knee arthrotomy, as well as revision cases, were excluded. The included patients were divided into 2 groups according to the applied pain management (group 1 FNB, group 2 LIA). Concerning the LIA group, a modified form of composition compared to the first describer without the use of adrenaline was carried out. Post-operative additional pain medications were given on a fixed scheme to the patient. The primary outcome was pain at rest over 7 days after surgery labeled by the numeric pain rating scale (NRS). The secondary outcome measures were the total amount of opioid consumption over the hospital stay and the additional need for non-opioid medication. The conversion of the opiate medications on the morphine preparation was carried out according to the conversion data from the literature. For functional recovery, we compared the range of motion in both groups, which was recorded from the second postoperative day by the attending physiotherapist.

**Results:**

In total, 202 patients were assessed for eligibility and included in this clinical trial. Hundred patients were allocated to the continuous FNB group (group 1) and 102 patients to the LIA group (group 2). No statistical difference was found between the two groups regarding demographic data. Primary outcome measurements: The LIA group had a significantly lower NRS score than the continuous FNB group for the measurement in the morning on days 1, 2, and 3 after surgery (day 1, 1.5; day 2, 1.6; day 3, 1.3; *p* < 0.05). Secondary outcome measurements: The total volume of morphine consumption for the first six postoperative days was significantly lower in the LIA group than the FNB group (FNB 159.8 vs. LIA 96.07). There is also a significant difference between the total morphine consumption of both groups in the direct postoperative course with respect to time and group (two way ANOVA, *p* < 0.05) On the day of the operation and on the first postoperative day, the intake of additional non-opioids in the LIA group was also significantly reduced compared to the FNB group. No significant difference was observed on the second to sixth postoperative day concerning an additional consumption of non-opioid medications. In terms of range of motion, the LIA group showed a higher active range of motion at the operated extremity than the FNB group during the hospital stay.

**Conclusion:**

The local intraarticular infiltration therapy (LIA) is a sufficient alternative to regional anesthesia avoiding the known risks of regional procedures. The results of this study reflect the efficiency of this pain management with a lower consumption of analgesics, identical to reduced postoperative pain ratings and an improved ROM in the first postoperative days.

**Level of evidence:**

Retrospective trial

## Introduction

Effective postoperative pain therapy and early mobilization has a significant influence on patient’s satisfaction and the spectrum of complications after implantation of a knee arthroplasty (TKA). A proven and effective principle is to perform regional anesthesia using an isolated and combined femoral and/or sciatic nerve block (FNB or SNB) with or without a permanent catheter [7, 8, 10, 11, 15, 18]. A combined pain medication therapy with opioid analgesics and non-steroidal anti-inflammatory drugs (NSAIDs, cyclooxygenase cox inhibitors) can be performed. Potential lowering of quadriceps strength, risk of injury of femoral nerve and infection, and need of special training and ultrasound equipment are some of the known risks of regional procedures. Further side effects by additional pain medication can be sedation, nausea, vomiting, and urinary retention in case of opioid usage. Non-selective cox inhibitors may cause gastrointestinal bleeding and renal complications [1, 2].

Kerr et al. initiated an alternative procedure in 2005 by introducing a local intraarticular infiltration therapy (LIA) with a combination of ropivacaine, ketorolac, and adrenaline, avoiding the mentioned risks of regional procedures [1, 3, 12]. Recent studies recommend to omit adrenaline from the LIA mixture in order to prevent possible negative side effects like tissue necrosis. Single-shot LIA with ropivacaine alone seems to result in clinically acceptable adequate pain control and can be easily used in daily TKA practice [17]. Furthermore, studies have shown that the LIA technique reduces the additional requirement for postoperative analgesia with opioids and can possibly lower the unwanted side effects [1, 4, 19, 21].

The aim of our study was to show that the use of a modified local intraarticular infiltration is an adequate alternative to standardized postoperative pain management, assuming the indicated advantages, in a large patient population. On the other hand, we want to prove that earlier postoperative mobilization with increased progress of range of motion as well as reduced additive use of painkillers within the first postoperative week is possible by performing LIA. We retrospectively compare two patient populations with LIA or a three-in-one femoral nerve block as selected pain management.

## Materials and methods

The study was approved by the regional ethics committee on 15 March 2012 (ref: 12/NW/0153). It was registered with controlledtrials.com, identifier: ISRCTN42045594. We conducted a non-randomized, non-blinded retrospective trial that included all patients that were scheduled to undergo primary unilateral TKA (TKA model Nex Gen LPS Flex Zimmer ®) by one single surgeon (R.H.) in a high-volume arthroplasty center between January 2015 and November 2015. The inclusion criteria were any patient undergoing a primary TKA for primary or secondary knee osteoarthritis without previous knee arthrotomy. Exclusion criteria were based on patients that lacked capacity to consent to the study or patients who were unwilling to consent and patients with known allergy to any of the drugs being administered. Patients who have secondary osteoarthritis due to rheumatoid arthritis or previous knee arthrotomy, as well as revision cases, were also excluded.

All surgeries were performed without a tourniquet with the medial parapatellar approach. The postoperative rehabilitation regimens were the same for both groups and started the day after surgery. Transfer to wheelchair and quadriceps setting exercise were started 1 or 2 days after surgery. Ambulatory training with weight bearing as tolerated and active-assisted range of motion exercise were started 1 day after surgery. Gait training using a walker was started at 2 days after surgery and using a cane at 3 days after surgery. Staircase climbing exercise and walking outside were started 3 days after surgery.

Demographic data as age, sex, height, weight and the resulting body mass index, operated side, and ASA Score were protocolled.

The included patients were divided into 2 groups according to the certain pain management, which was performed. Group 1 was treated as described in Table 1 with a three-in-one femoral block. The procedure was carried out according to the standardized protocol for performing an ultrasound-guided nerve block. The starter dose contained ropivacaine 0.2% 20 ml and prilocaine 1% 20 ml. A maintenance dose of 0.2% 20 ml was given every 6 h up to a maximum of 72 h after surgery. Post-operative pain medication as ibuprofen, oxycodone, and metamizole were given on fixed scheme to the patient.

**Table 1:** Pain protocol for group 1 (FNB group)

Femoralis-block 3 in 1 (group 1)	
**Postoperative pain medication:**	
Ibuprofen 600 mg 1-1-1 per os	
Metamizole 500 mg 1-1-1 per os	
Oxycodone 10 mg = 1-0-1 (3 days), per os	
**Pain catheter:**	
At posing: ropivacaine 0.2% 20 ml + prilocaine 1% 20 ml intravenous	
Every 6 h up to max. 72 h post-surgery: ropivacaine 0.2% 20 ml intravenous	

Group 2 was treated according to the protocol of Table 2 with the LIA scheme. Here, we worked with a modified form of composition compared to the first describer without the use of adrenaline and injectable NSAID (e.g., ketorolac). No tourniquet was used intraoperatively. Preoperatively, 2 g (i.v) and 3 g tranexamic acid (i.a.) right after capsule closure were injected. After irrigation and drying of the knee, the first syringe with 50 ml ropivacaine (2% ropivacaine/20 ml) was performed in the posterior capsule before cementing the prosthesis. After cementing both components and placing the final insert, the second syringe with 50 ml ropivacaine (2% ropivacaine/20 ml) was injected. The third and last syringe with 50 ml ropivacaine (2% ropivacaine/20 ml) was injected after closing the capsule into the subcutaneous tissue and skin. Postoperative pain medication on intermediate care and regular ward included oxycodone, naproxen, paracetamol, and gabapentin in a fixed scheme Table 2.

**Table 2:** Pain protocol for group 2 (LIA group)

LIA-Schema (group 2)	
**Premedication (if no known contraindications) 2 h preoperatively**	
Pantoprazole 40 mg per os	
Naproxen 500 mg per os	
Gabapentin 300 mg per os	
Paracetamol 1000 mg per os	
**Intraoperative infiltration of surgical area with ropivacaine 0.2% done by the surgeon**	
(3 × 50 ml with ropivacaine 0.2% 20 ml and 30 ml NaCl 0.9%)	
TIVA or spinal anesthesia	
**Postoperative pain therapy on the intermediate care–(if no known contraindication)**	
Oxycodone 10 mg = 0-0-1 per os (3 days)	
Naproxen 500 mg = 0-0-1 per os starting at 8 pm of the day of surgery	
Paracetamol 1000 mg—every 6 h iv	
**Postoperative pain therapy on regular ward (if no known contraindications)**	
Oxycodone 10 mg = 0-0-1 per os	
Gabapentin 300 mg = 1-0-0 per os	
Naproxen 500 mg = 1-0-1 per os	
Paracetamol 1000 mg = 1-1-1-1 per os	

In both groups, additional pain medication according to the WHO pain ladder was prescribed on patient’s requirements (level 1: diclofenac, ibuprofen, paracetamol, or metamizole; level 2: level 1 + tramadol po or iv; level 3: level 1 + oxycodone po, piritramide sc/iv or pethidine iv).

The two protocols are slightly different from each other due to the fact that when switching to the LIA scheme, we applied changes to the pain protocol based on recent literature. Giving pain medication already before the surgery allowed us to reduce the standard doses after the surgery. Being a retrospective trial and comparing our modifications to our previous pain protocol, we are aware of this study limitation.

The primary outcome was pain at rest over 7 days after surgery. For the objective measurement of pain intensity, the numeric pain rating scale (NRS) was used. Patients were asked to quantify their pain on a scale of 0 to 10, while 0 means no pain and 10 the strongest imaginable pain. Immediately after the operation in the intermediate ward, the nurse asked the patient to quantify his pain intensity. Since the operations were performed at different times of the day, the measurement of pain intensity was not related to a particular daytime. On the regular ward, the pain was measured in the morning and evening throughout the hospital stay. The measurement refers to the intensity of resting pain. The postoperative pain scores were taken from the patient’s personal file, and the mean values were adopted for a comparison between the two groups.

The secondary outcome measures were the total amount of opioid consumption over the hospital stay and the additional need for non-opioid medication, evaluated by the patient’s file. The conversion of the opiate medications on the morphine preparation was carried out according to the conversion data from the literature Table 3.

**Table 3:** Representation of the conversion factors for the equivalent dose of 1mg morphine

tramadol	po	x0.20	morphine po 1g
pethidine	iv	x0.13	morphine po 1g
oxycodone	po	x1.50	morphine po 1g
piritramide	iv	x0.66	morphine po 1g
piritramide	sc	x0.66	morphine po 1g

For functional recovery, we compared the range of motion, which was recorded from the second postoperative day by the attending physiotherapist using a goniometer. The total extent was measured according to the extension and flexion.

Statistical analysis was carried out using the SPSS software (SPSS Inc, Chicago, IL). The control of the variance equality was performed according to the Levine test. The chi-square independence test was performed to compare categorical variables. The independent *t* test was used to compare continuous variables after determining the distribution was appropriate for parametric testing. As the data from this study were not to be normally distributed, the Mann–Whitney *U* test was used for analysis. *P* values < 0.05 were considered significant.

## Results

In total, 202 patients were assessed for eligibility and included in this clinical trial. Hundred patients were allocated to the continuous FNB group (group 1) and 102 patients to the LIA group (group 2). Table 4 summarizes the demographic characteristics of the two patient groups without showing any significant differences.

**Table 4:** Demographic data of the analyzed patients

	FNB group I	LIA group II	***P*** value
	(***n*** = 100)	(***n*** = 102)	
**Age** (year)	70 ± 8	73 ± 9	0.04^1^
**Sex** (female/male, %)	56/44	57.8/42.2	0.89^3^
**BMI** (kg/m^**2**^)	28.6 ± 4.3	27.6 ± 5.4	0.08^2^
**Operated side** (left/right, %)	49/51	50/50	0.89^3^
**ASA score**	1.97 ± 0.7	2.14 + 0.6	0.32^3^

Results are expressed as mean ± standard deviation, unless stated otherwise. *ASA* American Society of Anesthesiologists classification; *BMI b*ody mass index, *p* values were determined with ^1^Students *t* test, ^2^Mann-Whitney-U-Test, ^3^Chi-Quadrat according to Pearson

### Primary outcome measure

**Fig. 1:**
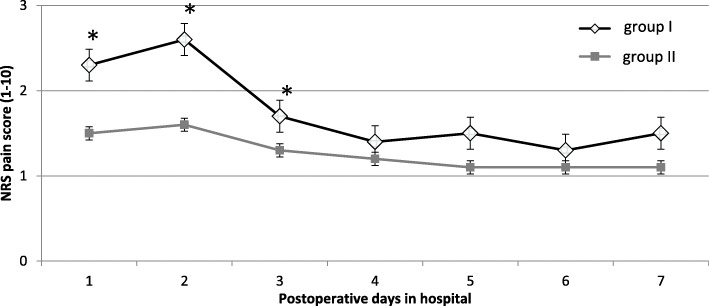
The pain scores at rest (mean and standard deviation) after total knee arthroplasty, as rated on 10 numeric pain rating scale (NRS) measured in the morning. The local infiltration analgesia group exhibits a significantly lower NRS score 1 day to 3 days after surgery in the morning (Mann-Whitney *U* test, *p* < 0.05*)

**Fig. 2:**
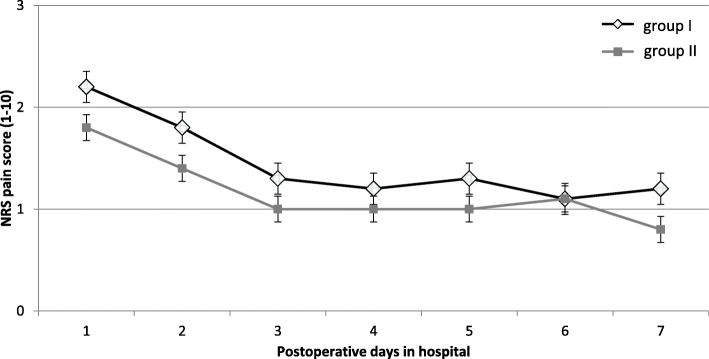
The pain scores at rest (mean and standard deviation) after total knee arthroplasty, as rated on 10 numeric pain rating scale (NRS) measured in the evening. No significant differences over the stay in hospital between both groups (Mann-Whitney *U* test)

The NRA scores at rest are shown in Fig. 1 (morning) and Fig. 2 (evening). In both groups, the hypotheses of normality were rejected according to the Kolmogorov-Smirnov test.

The LIA group had a significantly lower NRS score 1 day after surgery than the continuous FNB group (mean 1.5 ± 1.1 vs 2.3 ± 1.6; *p* < 0.05) for the measurement in the morning (Fig. 1). A significant difference between the two groups could also be observed on the second and third postoperative day (day 2: mean 1.6 ± 1.2 vs 2.6 ± 1.7; *p* < 0.05; day 3: mean 1.3 ± 1.3 vs 1.7 ± 1.2; *p* < 0.05). From the fourth to the sixth postoperative day, no significant difference in pain intensity was shown for the morning measurements. On the seventh postoperative day, there was again a significant difference between the two groups (day 7: 1.1 ± 1.1 vs. 1.5 ± 1.2, *p* < 0.05). Figure 2 reflects the evening results without evidence of a significant difference between both groups over time.

### Secondary outcome parameter

Concerning pain medication consumption, there is a different basis for opiate therapy in terms of drug-based pain therapy due to the retrospective nature of the study. For a better comparison, the conversion of the opiate medications on the morphine preparation was carried out according to the conversion data from the literature (Table 3). The original drug consumption without conversion is shown in Table 5.

**Table 5:** Presentation of opiate medications per day and group and patient in mg without conversion

Pain medication_group		Number of patiens (*n*)	Pain medication/day/patient (mean)	Pain medication/day/patient (SD)
Tramadol_day of surgery (mg)	Femo	100.00	0.00	0.00
	LIA	102.00	0.74	7.43
Tramadol_day 1 (mg)	Femo	100.00	4.50	19.91
	LIA	102.00	6.13	27.80
Tramadol_day 2 (mg)	Femo	100.00	0.00	0.00
	LIA	102.00	2.94	18.04
Tramadol_day 3 (mg)	Femo	100.00	0.00	0.00
	LIA	102.00	1.23	8.88
Tramadol_day 4 (mg)	Femo	100.00	0.00	0.00
	LIA	102.00	0.00	0.00
Tramadol_day 5 (mg)	Femo	100.00	0.00	0.00
	LIA	102.00	0.74	7.43
Tramadol_day 6 (mg)	Femo	100.00	1.50	10.55
	LIA	102.00	1.47	12.60
Oxycodone_day of surgery (mg)	Femo	100.00	4.60	6.10
	LIA	102.00	8.73	3.35
Oxycodone_day 1 (mg)	Femo	100.00	17.80	9.05
	LIA	102.00	11.47	6.20
Oxycodone_day 2 (mg)	Femo	100.00	18.20	8.33
	LIA	102.00	9.31	7.07
Oxycodone_day 3 (mg)	Femo	100.00	15.70	9.13
	LIA	102.00	7.16	6.80
Oxycodone_day 4 (mg)	Femo	100.00	13.30	9.65
	LIA	102.00	5.20	6.41
Oxycodone_day 5 (mg)	Femo	100.00	12.40	10.26
	LIA	102.00	4.71	6.40
Oxycodone_day 6 (mg)	Femo	100.00	10.90	11.29
	LIA	102.00	5.98	7.35
Piritramide_day of surgery (mg)	Femo	100.00	10.24	7.68
	LIA	102.00	10.77	8.14
Piritramide_day 1 (mg)	Femo	100.00	5.14	6.28
	LIA	102.00	5.55	7.02
Piritramide_day 2 (mg)	Femo	100.00	3.38	5.58
	LIA	102.00	1.51	3.31
Piritramide_day 3 (mg)	Femo	100.00	1.65	4.21
	LIA	102.00	0.51	2.18
Piritramide_day 4 (mg)	Femo	100.00	1.65	4.59
	LIA	102.00	0.59	2.52
Piritramide_day 5 (mg)	Femo	100.00	1.88	5.68
	LIA	102.00	0.51	2.84
Piritramide_day 6 (mg)	Femo	100.00	2.18	5.57
	LIA	102.00	1.10	4.00
Pethidine_day of surgery (mg)	Femo	100.00	15.50	22.69
	LIA	102.00	9.07	17.87

**Fig. 3:**
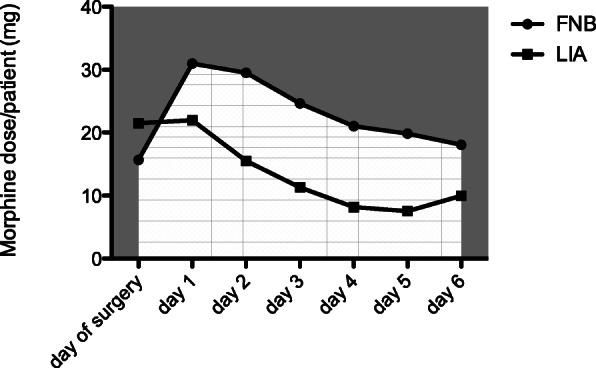
Graphical representation of total opiate consumption over the entire inpatient stay. There is a significant difference between the groups over time. The total dose/patient/7d is also significantly different in favor of the LIA group

With regard to pain medication, after conversion to a morphine equivalent, there is a significant difference between the total morphine consumption of both groups in the direct postoperative course with respect to time and two-way ANOVA (group 1 22.83 mg vs. group 2 13.72 mg (*p* = 0.01)). Relative to the comparison between the two groups on the respective postoperative day, the day of the operation shows a decent higher, not significant consumption (21.52 mg ± 5.98 (LIA) vs 15.67 ± 3.46 mg (FNB)). In the following postoperative days 1–6, there is a higher but non-significant use of opiates in the FNB group (day 1, 30.99 mg ± 14.23 vs. 21.99 mg ± 8.55/day 2 29.53 mg ± 15.16 vs. 15.53 mg ± 7.6/day 3 24.64 mg ± 13.29 vs. 11.32 mg ± 6.03/day 4 21.04 mg ± 11.22 vs. 8.18 mg ± 4.39/day 5 19.84 mg ± 10.4 vs. 7.54 mg ± 3.94/day 6 18.09 mg ± 8.95 vs. 9.99 mg ± 4.89) (Fig. 3).

**Fig. 4:**
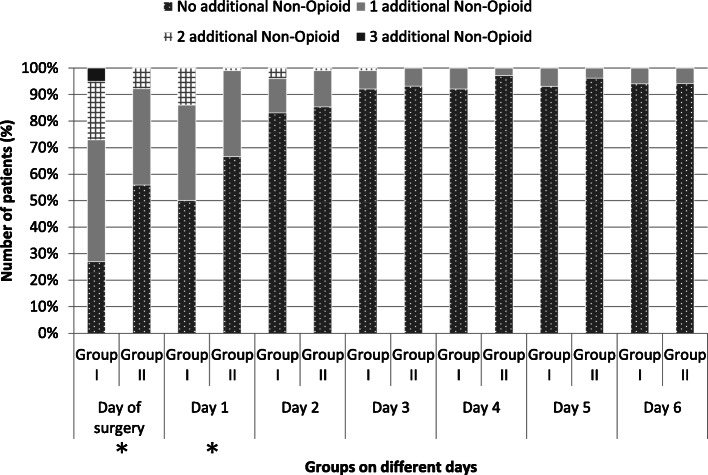
This graph shows the different additional consumption of the non-opioid medication for the first 6 postoperative days between both groups. Results are expressed as number of patients in percentage. *p* values were determined by Mann-Whitney *U* test, *p* < 0.05*)

Additionally Fig. 4 shows the comparison of the two groups for the use of additional non-opioid drugs during the first 7 days of hospitalization. On the day of surgery, 27% of the FNB group received no additional non-opioid medication, 46% an additional, 22% two additional, and 5% received three additional non-opioids. In contrast in the LIA group 56% received no additional non-opioid medication on the day of surgery, 36% an additional, and 8% two additional non-opioid medications. In summary, the FNB group needed significantly more additional non-opioid medication on the day of surgery (*p* < 0.05). Similar results could be shown on the 1st postoperative day, where 50% in the FNB group received no additional non-opioid medication, 36% an additional, and 14% two more non-opioid medications. In contrast, in the LIA group, 67% did not need any additional medication, 32% received one, and only 1% two more non-opioid medications. The difference between both groups was likewise significant (*p* < 0.05). No significant difference was observed between the two groups on the second, third, fourth, sixth and seventh postoperative day concerning an additional consumption of non-opioid medications (*p* > 0.05).

In terms of range of motion on each postoperative day, a significant difference in movement between the two groups was observed. The LIA group showed a higher active range of motion at the operated extremity than the FNB group. Although there is a significant difference over the entire included observation period, the largest numerical difference is in the first 3 days and the movement rates approach until the 4th postoperative day (Table 6).

**Table 6:** Range of motion postoperative days 2–7 of the analyzed patients

Postoperative day	FNB group	LIA group II	***P*** value
	(mean, SD)	(mean, SD)	
Day 2	36° ± 7	46° ± 10	< 0.05^1^
Day 3	46° ± 8	55° ± 10	< 0.05^1^
Day 4	56° ± 8	63° ± 11	< 0.05^1^
Day 5	64° ± 9	69° ± 11	< 0.05^1^
Day 6	70° ±	74° ± 12	< 0.05^1^

Results are expressed as mean ± standard deviation. *p* values were determined by ^1^Students *t* test

## Discussion

Effective pain therapy, especially for direct postoperative pain reduction, has a significant influence on patient satisfaction and the spectrum of complications after implantation of a knee arthroplasty (TKA). A proven and effective principle is to perform regional anesthesia using an isolated and combined femoral and/or sciatic nerve block (FNB or SNB) with or without a permanent catheter. As an alternative procedure, we investigated the local intraarticular infiltration therapy (LIA) avoiding the known risks of regional procedures in this study with a modified drug mixture. At present, the most effective pain management continues to be the subject of controversy.

The results of this work reflect the efficiency of this pain management with a lower consumption of analgesics, identical to reduced postoperative pain ratings and an improved ROM in the first postoperative days.

Further recent works has demonstrated a possible equivalent pain management using the LIA method, whereby the individual composition of substances such as adrenaline or different cortisone preparations still varied. Since LIA affects only the surgical area with moderate analgesia, there is limited interference to muscle strength of the lower limb and safety in short-time follow-up [14]. Besides a low infection rate, the procedure can be done by surgical team; no special skills are required to administer an injection into the periarticular tissues. Because the quadriceps strength is not inhibited, there is no motor paralysis caused. This means that the legs can be moved voluntarily from the early period after surgery, reducing the deep vein thrombosis associated with venous stasis.

In our study, we compared this 2 routinely used methods that are both effective for postoperative pain relief in TKA. The large number of procedures for each pain management performed at one hospital by one high volume surgeon (LIA = 102 vs. FNB = 102) is methodologically advantageous. Concerning the outcome parameter pain intensity, pain killer consumption, and postoperative range of motion, a slight superiority of the local infiltration method over the singular femoral catheter is shown. Up to 72 h after surgery, there was a significant reduction of the NRS score in the recorded values from the 1st–7th postoperative day with advantage to the patient group with regional nerve blocks. With regard to opiate consumption, there is an increased consumption of painkillers in the patient group with FNB compared to the LIA patient population. The functional outcome parameter range of motion (ROM) also shows significantly better results for the 1st to 7th postoperative day after surgery, although a rapid approximation of the movement amounts is seen.

The present publication even tends to show an apparent superiority; however, this can only be expressed with reservations in the case of existing limitations of the study. These include the retrospective, non-randomized study design with consecutive influence on outcome parameters such as pain anamnesis, opiate consumption, and functional postoperative rehabilitation. The two patient groups (LIA vs. FNB) received different additive pain medications pre- and postoperatively, so that a direct comparison of drug consumption can only be carried out to a limited extent. Previous studies also showed a positive effect on pain reduction by addressing the posterior capsule with a combined procedure of FNB and SNB, which could lead to a renewed reduction of pain medication. For regional reasons, only FNB was used. The preoperative consumption of painkillers could no longer be determined retrospectively as an influencing factor on the later consumption. Missing outcome parameters such as length of hospital stay, side effects, and patient’s satisfaction with pain control are not documented and not subsequently evaluated.

Despite two current meta-analyses, the heterogeneity of the various, existing study designs makes it difficult to accurately assess the effectiveness of the intraarticular infiltration method. The definition of the outcome parameters differs in object and observed time span. Both the application of painkillers by catheter or single shot or in combination with additive regional procedures and the composition of the intra-articular pain medications are various.

In their meta-analysis, Zhang et al. evaluate the results of 10 publications (RCT) that compare the efficacy of LIA with peripheral nerve blocks, primarily FNB [22]. The outcome parameters are based on VAS (or NRS), total morphine consumption, ROM and length of hospital stay. The comparative analysis of the data showed a similar reduction of VAS after 24 h, 48 h, and 72 h postoperatively with reduction of morphine consumption and improved range of motion without increase of the complication spectrum. The VAS was the primary outcome parameter. Five studies including 231,535 patients reported VAS scores for postoperative day 1 to day 3 that showed no significant difference between both groups.

Fan et al. already showed similar results in a further meta-analysis (8 RCT), whereby 4 included publications were included in both meta-analyses [9]. Again, the heterogeneity of the studies as well as the factors influenced by bias such as emotions, threshold of pain, and sociocultural background were evaluated as decisive influences on the VAS (NRS).

On the other hand, Fan et al, in its previous meta-analysis, could show that NRS values for pain on rest were lower in the LIA group than in the group of regional methods applied. As to the NRS score with activity, there was no significant difference between the two groups.

Toftdahl et al. could not detect any significant difference directly postoperatively between LIA and FNB analgesic patients at rest until the 2nd postoperative day (pod), but there was a better pain reduction in the first 24 h postoperatively after LIA, if the patient has already been treated in physiotherapy [20]. Carli et al., however, showed significantly lower VAS values at rest in the LIA group and could not detect any difference between the two groups during physiotherapy [5]. Affas et al. postulated significantly better pain management at rest and in physiotherapy in the group of patients who received articular infiltration therapy [1]. In a recent study by Kurosaka et al., the LIA group showed superiority within the first 24 h without differentiation between rest and stress [13].

The outcome parameter of morphine consumption is influenced by the fact that different opiates have been applied pre- and postoperatively. A comparison of the publications was approximated by the conversion into equivalent doses. Zhang et al. showed no difference between the two groups, whereby no time scheduling is shown. In contrast, Fan et al. describes lower morphine consumption in the LIA group compared to the patient group of the regional procedures on the 1st postoperative day with a low heterogeneity factor in its pooled data. In most studies, including our own, there is reduced opiate demand in the first 24 h [1, 6, 13, 16, 20]. In contrast, Carli et al. showed in a double-blinded, randomized, controlled study that the group of FNBs were associated with a significantly reduced postoperative consumption of morphine and a trend to better analgesia compared with periarticular infiltration [5]. The fact that in this study the FNB was only used in combination with a sciatic nerve block served as an explanation.

The comparison of both treatment strategies on the basis of the outcome parameter of postoperative functionality is again made difficult by the decisive differential definition of an acceptable function. Zhang et al. evaluated the passive ROM in 4 studies in 308 patients. There was no significant difference between the FNB and LIA group with regard to the knee ROM. Fan et al. also with reference to a high heterogeneity factor, also could not detect any significant difference in the range of motion after 3 months postoperatively. However, the study indicates earlier mobility and shorter hospital stays in the group of LIA patients [6, 20].

Our data indicate that the 2 analgesic regimens gave similar quality of pain relief and time of functional recovery during the directly postoperative time. Both FNB and LIA resulted in low average pain intensity. A femoral nerve block is commonly administered to reduce the side effects and complications related to self-administered analgesia in patients who have had a TKA. Nevertheless diminished muscle control, nerve damage, and local infection are recognized complications, ranging from 0.1 to 2.5%, and 15% of femoral nerve blocks are unsuccessful. LIA offers the benefits of blocking pain influx at its origin and maximizing muscle control.

Both procedures are methods of analgesia that have an excellent effect for pain management after TKA, but the types, doses, and methods of administration of the agents used have yet to be established. Despite existing meta-analyses including high-quality studies (prospective, blinded design), the sample size of all included studies is very small. With 101 patients per treatment branch for pain management, our study confirms the efficacy of intra-articular infiltration therapy as an attractive alternative treatment method without the risks of regional procedures.

## Conclusion

The LIA group showed in this study a significantly lower consumption dose of opioid and a quicker recovery of range of motion than the FNB group. Furthermore additional pain killer medicine could be reduced during the hospital stay. The LIA group proved to be as effective as the FNB group.

## Data Availability

All data generated or analyzed during this study are included in this published article. The datasets generated during and/or analyzed during the current study are also available from the corresponding author on reasonable request.

## Abbreviations

LIA: Local infiltration analgesia
FNB: Femoral nerve block
TKA: Total knee arthroplasty
NRS: Numeric rating scale
SNB: Sciatic nerve block
NSAIDs: Nonsteroidal anti-inflammatory drugs
COX: Cyclooxygenase
ASA: American Society of Anesthesiologists
e.g.: Exempli gratia
i.v.: Intravenous
i.a.: Intra-articular
p.o: Per os
RCT: Randomized controlled trial
VAS: Visual analog scale
pod: Postoperative day
ROM: Range of motion
